# Hospital Porters and Solicitors' Touts

**Published:** 1913-01-04

**Authors:** 


### HOSPITAL PORTERS AND SOLICITORS' TOUTS.

For a long time—in fact, ever since the first of the Workmen's Compensation Acts—an evil has been growing up which has already reached serious proportions. We refer to the mischief, so ably demonstrated by Sir John Collie in a recent letter to the *Times*, of speculative and fraudulent claims for damages after trivial injuries and accidents, engineered and fomented by shady solicitors. This practice, to which we referred more than once in April last, breeds an amount of malingering, deceit, and dishonesty which is rapidly on the increase. It is the direct cause of a great deal of neurasthenia and demoralisation, and indirectly of alcoholism and even death, for successful claimants only too often celebrate the occasion in a way which leads to dissolute habits.

Sir John's trenchant exposure of the methods of the swindling solicitors and the touts upon whom they saddle the responsibility if they are found out must be read to be appreciated, and it well repays careful study. From the institutional point of view the interest lies mainly in the devices adopted for securing the names and addresses of those who after accidents become patients in hospitals. Sir John believes it is often done by waylaying relatives or friends on visiting day, and doubtless he is right. There is, however, another avenue of approach which is a good deal used, we are sorry to say. In not a few cases the names of injured persons are secured by bribing the porter or porters of the hospitals. It is nobody's business, apparently, to put any check on this sort of illicit commissions, and the facts probably would in any case be difficult to prove. But unquestionably the practice prevails; medical superintendents and secretaries might do worse than pay it serious attention. By interesting the house surgeons in the quest, evidence would soon accumulate if the matter were systematically investigated, and the summary measure of dismissal without a character would be no more than justice for any employee at a hospital against whom a charge of this nature could be proved.

We have thought it our duty to state these unpleasant matters plainly, since an evil exists which is in urgent need of drastic remedies. Let us at once add that we are very far from intending to cast a slur upon the general body of hospital porters or any other class of institutional worker. In this, as in other cases, the many have to suffer in reputation for the faults of the few, and, indeed, we should hardly credit the corruption we have described had we not excellent grounds for knowing that it exists beyond cavil. The temptation is offered in the first place by the touts acting as agents for the dishonest solicitors who batten on this traffic, and the major portion of the guilt is theirs. With them we have, of course, no immediate concern, but we very strongly support Sir John Collie's appeal to the Incorporated Law Society to do something in the way of penalising these rogues, whose methods are apparently winked at by the official governing body of the legal profession. The appointment of a poor man's lawyer has, Sir John states, now become a necessity, and he suggests that men of integrity should be appointed by the Government for such purposes. We recommend the suggestion to the Chancellor, who might be able to tack it on to the Insurance Act on a "panel" basis.

